# “It’s Healthy Because It’s Natural.” Perceptions of “Clean” Eating among U.S. Adolescents and Emerging Adults

**DOI:** 10.3390/nu12061708

**Published:** 2020-06-07

**Authors:** Suman Ambwani, Gina Sellinger, Kelsey L. Rose, Tracy K. Richmond, Kendrin R. Sonneville

**Affiliations:** 1 Department of Psychology, Dickinson College, P.O. Box 1773, Carlisle, PA 17013, USA; 2 Department of Nutritional Sciences, University of Michigan School of Public Health, Ann Arbor, MI 48109, USA; gselling@umich.edu (G.S.); klserose@umich.edu (K.L.R.); kendrins@umich.edu (K.R.S.); 3 Division of Adolescent Medicine, Boston Children’s Hospital, Boston, MA 02115, USA; tracy.richmond@childrens.harvard.edu

**Keywords:** eating, diet, text-messaging, disordered eating, adolescents, emerging adults, awareness, attitudes

## Abstract

Definitions for the culturally trendy “clean” eating phenomenon vary: whereas some characterize it as natural and healthy, others adopt more restrictive, moralizing, and affectively-laden definitions that may reflect disordered eating. We examined levels of familiarity with “clean” eating, sources of information, and perceptions of this dietary trend among a large, diverse sample of U.S. adolescents and emerging adults recruited from the National MyVoice Text Message Cohort (*n* = 1266; ages 14–24 years). Participants answered five questions assessing knowledge of “clean” eating, definitions, perceived healthiness vs. harm, and willingness to adopt “clean” eating, and responses were coded by three trained researchers. Results indicate that 55% of respondents had previously heard of “clean” eating, most commonly through social media, other online sources, and peers. Definitions were heterogeneous, with 40% offering “non-processed” or “whole foods” and 13% noting “non-GMO” or “organic” components. Few respondents (0.6%) expressed outright skepticism about “clean” eating, but many (30%) identified dietary avoidance and restriction as part of the definition. Overall, 71% characterized “clean” eating as a healthy approach, whereas 6% flagged it as “unhealthy”, and 18% noted elements of both healthfulness and harm. Notably, 41% reported they “probably would” try “clean” eating themselves, with greater willingness to try “clean” eating among cisgender women. Present findings highlight high levels of awareness and positive attitudes toward “clean” eating among young people in the U.S., with little recognition of the potential risks of dietary restriction. Further research should examine actual dietary behaviors to clarify potential risks of “clean” eating and related trends and thus inform strategies for eating disorder prevention.

## 1. Introduction

Dieting is a popular endeavor, particularly when it is framed as a health pursuit; a 2019 survey of over 1000 U.S. adults indicated that 38% had followed a diet over the past year, of which “clean” eating was the most commonly cited diet [1]. “Clean” diets and labels are heterogeneous entities, without clear definitions or oversight from regulatory authorities, resulting in their broad usage by industry and varied interpretation by consumers [2]. For instance, whereas some “clean” diets emphasize the consumption of whole, unprocessed foods, others involve eliminating entire food groups such as dairy, refined sugar, wheat, and “alkaline” foods [3,4]. While “clean” recipes may be no more nutritious than matched control recipes [5], the pursuit of “wellness” through “clean” eating is closely tied to the values of healthism, an approach that singularly focuses on individual responsibility to pursue health [3]. Although pursuers of “wellness” benefit from social acceptance and ‘moral citizenship’ [6], the cultural moralization of eating behavior, signaled by the ambiguous language of “clean” (vis à vis “dirty”) foods could increase harmful dietary restriction and preoccupation with so-called “healthy” eating among vulnerable groups including individuals at risk for eating disorders. Furthermore, cultural preoccupations with “clean” eating may promote dichotomous thinking, an approach characterized by extreme “all bad” or “all good” views toward food.

Although dieting is a well-established risk factor for disordered eating in adolescents and young adults [7,8,9,10,11,12], research linking the two has historically focused on diets explicitly branded for weight loss. However, research also suggests that following a special diet (e.g., vegan/raw, paleo, gluten-free) may be linked with higher rates of eating disorders [13], evidence that is particularly salient within the backdrop of a burgeoning wellness industrial complex [14,15]. Nonetheless, the impact of new waves of “wellness” focused diets remains poorly understood.

“Clean” diets may require particular scrutiny for their ability to mask eating disorder symptoms as ostensibly “healthy” behaviors and thereby prevent detection and intervention [16]. Indeed, research suggests that positive attitudes toward “clean” diets are linked with disordered eating attitudes and behaviors [4] and those who followed advice from “clean” eating websites exhibited higher dietary restraint, a risk factor for disordered eating [17]. Although not yet recognized as a standalone diagnosis, research suggests that orthorexia nervosa (ON) reflects an unhealthy preoccupation with healthy eating, one associated with substantial distress and functional impairment mirroring eating disorder psychopathology [18,19]. Moreover, the National Eating Disorder Association suggests that the “clean” eating trend may be associated with ON [20], and scholars often describe ON as an extreme variant of “clean” eating (e.g., [6,21,22]). Thus, “clean” eating may involve excessive dietary restriction resembling an eating disorder [23] and thereby increase risk for nutritional deficiencies [24]. Even more concerning is the potentially expansive reach of “clean” diets: individuals who may not have been susceptible to dietary restriction for weight loss may be vulnerable to new diet approaches promoting the obsessive pursuit of wellbeing [3]. For instance, a recent pilot investigation reported generally favorable perceptions of “clean” diets among a small sample of U.S. undergraduates, even when those diets were linked with substantial distress and dysfunction [4]. Thus, these new dietary approaches need to be better understood, particularly among a larger swath of adolescents and young adults, in order to facilitate macro-level eating disorder prevention, a key priority for eating disorder research [25,26].

Whereas research suggests that adolescents and emerging adults are particularly susceptible to dieting and disordered eating [11], perceptions of “wellness” oriented diets among adolescents and emerging adults remain poorly understood. Further understanding of how young people view “clean” eating could inform prevention and intervention efforts to reduce risk for nutritional deficiencies and disordered eating and thereby promote public health. Thus, the purpose of the current exploratory study was to examine impressions of “clean” eating and gender differences in attitudes toward “clean” diets among a large, diverse sample of young people in the United States.

## 2. Method

### 2.1. Participants

Participants were from the MyVoice Text Message Cohort (*n* = 1266 respondents; [27]). A group of individuals aged 14–24 years were recruited via targeted Facebook and Instagram ads to match national demographics based on weighted samples from the 2016 American Community Survey (ACS). Demographic features for those who responded to at least one question on the current survey (*n* = 1020) are reported in Table 1. This study was approved by the University of Michigan Institutional Review Board (HUM00119982). Participants provided consent online and were compensated $1 for responding to the survey.

### 2.2. Measures

Demographics. Participants self-reported age, gender, race, zip code, marital status, education level (self and parent/guardian), and socioeconomic status as part of their registration in the MyVoice cohort. We did not assess current dietary behaviors. Additional details about the cohort have previously been published [27].

Perceptions and attitudes toward “clean” eating questions. Participants were prompted to answer five predominantly open-ended questions about “clean” eating, described as “an approach to eating that has been getting a lot of attention lately.” Questions were presented individually in the following order and could not be answered retroactively: (1) Have you previously heard about “clean” eating? If so, how did you hear about it? (2) How would you define “clean” eating? (3) “Clean” eating usually means eating natural, whole foods, and strictly avoiding processed foods. Knowing that, what is your opinion of “clean” eating? (4) Do you think “clean” eating is healthy or harmful? Tell us why. (5) How likely would you be to try “clean” eating for yourself? (1 = probably will not try it; 2 = not sure; 3 = probably will try it). Questions were pilot-tested with the MyVoice team prior to administration.

### 2.3. Procedure

Participants responded to questions via text message between January–February 2019. Due to the carefully curated nature of the MyVoice cohort and the use of captcha and text message response procedures for providing consent [27], there were no concerns about random responding by bots. All responses were checked by the primary author (S.A.) to exclude nonsensical or implausible answers (e.g., strings of letters or digits, random answers that did not match the questions) and correct any misalignments in data due to the text messaging system (e.g., long responses that might have carried over to the next column of data). After data cleaning, the number of total respondents varied by question (*n* = 945–1020).

### 2.4. Data Analyses

For the open-ended questions, length of responses varied from 2 to 967 characters, with specific character counts per question as follows: question 1 (M = 32.35, SD = 36.22), question 2 (M = 54.47, SD = 40.49), question 3 (M = 75.92, SD = 69.35), and question 4 (M = 93.26, SD = 76.27). We used qualitative content analysis to code responses to these questions to capture nuances and identify distinct themes based on the data [28]. Analysis proceeded in four phases. First, the primary author (S.A.) generated initial themes based on reviewing the first 100 responses for each question. The investigative team (S.A., K.R.S., T.K.R.) then reviewed those data to generate additional themes and reached a consensus on primary themes. Second, the themes were clarified and revised slightly based on feedback from coding 25 additional responses by three coders (K.L.R., G.S., H.S.). Third, these coders independently coded the responses for each question in the data set (responses could be coded for multiple themes). Inter-coder reliability was determined by calculating agreement between all three coders. A total of 151 responses (~15% of total responses) were triple-coded, at which point agreement statistics consistently indicated over 90% agreement. Fourth, two coders (K.L.R., H.S.) independently coded the remaining responses and the third coder (G.S.) received the double-coded responses in batches, calculated agreement at each round, and provided continuous clarification on the final themes. Final estimates for interrater agreement ranged from 78–99% for each theme (M = 94.37%; SD = 0.06) and discrepancies were resolved by the third coder. We conducted frequency analyses (counts and percentages) to summarize the occurrence of various themes in participant responses. We then conducted exploratory chi-squared analyses of independence to compare interest in “clean” diets across gender categories, followed by Bonferroni-corrected post-hoc analyses to compare those who were interested versus unsure or uninterested in trying “clean” diets.

## 3. Results

Among those in the MyVoice cohort, 1020 participants provided interpretable responses to at least one of the questions in the current survey (80.57% response rate). Over half of the respondents had heard about “clean” eating (54.6%), and they reported learning about it from a wide variety of sources, with social media most commonly specified (see Table 2).

Participant definitions of “clean” eating were heterogeneous, with many highlighting elements of perceived healthiness (38.7%), dietary restriction (29.7%), and processed or whole foods (39.7%), and few identifying weight loss goals (<1%), detoxification (2%) or outright skepticism about “clean” diets (<1%) as part of their definition (see Table 3). Participant opinions of “clean” eating included comments on its perceived healthiness (with 88.3% viewing it as healthy), impact on wellbeing (with 75% viewing it as having a positive impact), ambiguous opinions (of which 88% were positively hued), and ambivalent or neutral opinions (7.5%), and others highlighted financial considerations, feasibility, and environmental considerations in shaping their opinions (1.2–15.3%). When asked to identify whether “clean” eating was healthy or harmful, most respondents identified it as healthy (70.8%), but some participants identified elements of healthfulness and harm (18%), such as through increased risk for eating disorders. In terms of willingness to try “clean” eating, 24.1% (220/912) reported they probably would not try it, 41.2% (376/912) probably would, and 34.6% (316/912) were unsure.

A chi-squared analysis of independence comparing willingness to adopt “clean” diets across gender categories was significant, χ^2^ (6) = 34.56, *p* < 0.01, with 46.8% (249/532) of cisgender women, 37.6% (118/314) of cisgender men, and 13.6% (9/66) of those identifying with other genders reporting that they “probably would” try “clean” eating themselves. Post-hoc analyses using a Bonferroni correction compared respondents who “probably would” to those who would not or were unsure; results indicated significant differences between all gender groups, including, cisgender men and cisgender women (χ^2^ (1) = 6.84, *p* = 0.009)), cisgender women and other gender (χ^2^ (1) = 26.33, *p* < 0.001), and cisgender men and other gender (χ^2^ (1) = 14.05, *p* < 0.001), with greater willingness to try “clean” eating among cisgender women, followed by cisgender men, than their other gender counterparts. 

## 4. Discussion

Dietary fads such as “clean” eating have potentially wide-reaching public health implications, ranging from increased eating disorder risk [17] to the potential for nutritional deficiencies among vulnerable individuals [24]. Our findings suggest high levels of awareness, largely favorable impressions, and high levels of interest in pursuing “clean” eating in a large, diverse sample of U.S. adolescents and young adults. Although definitions varied widely, our data suggest that “clean” eating is frequently characterized by unprocessed or whole foods (such as raw foods, natural foods, or foods without artificial colors or flavors) and sometimes within the context of farming practices such as non-genetically modified (GMO), organic or environmentally-friendly/sustainable farming methods. These predominantly positive impressions may reflect the ways in which “clean” diets are marketed and the cultural moralization inherent in the language of clean/dirty. Moreover, a minority of respondents demonstrated awareness of the potential for harm conferred by “clean” eating pursuits. Public health messaging that highlights these risks may facilitate a more nuanced understanding about “clean” diets and related “wellness” health trends among a broader swath of young people.

Notably, very few participants identified weight loss or calorie reduction as key themes in their definition of “clean” eating, and although “clean” eating was frequently defined using language of dietary restriction, few respondents offered restrictive dietary behaviors as a potential harmful consequence of following “clean” diets. In a recent analysis of women bloggers’ experiences with orthorexia nervosa, participants reported that their diets were initially motivated by health problems (such as digestive issues) and a desire to be healthy, but that social influences (such as social comparisons of “healthiness”, and praise for pursuing “healthy” behaviors) and lack of awareness fueled their difficulties [22]. These data suggest that weight loss is likely not a defining feature for those who may eventually go on to pursue “clean” diets in an unhealthy manner, and that further research is needed to identify pathways that tip the balance from healthy to unhealthy pursuits.

Given recent calls to address macro-environmental factors for eating disorder risk [25,26], current findings signal a need to direct greater attention toward “clean” diets in prevention campaigns directed toward adolescents and young adults in the U.S. These results also suggest a need to promote social media literacy, which was identified as the top source of information for “clean” eating among our respondents. Indeed, social media is an important source of information about healthy eating (e.g., Facebook and Instagram are used to guide nutrition-related decision-making; [29]), but it is easy to imagine how social media promotion of “clean” eating can be harmful. For instance, one participant in a qualitative investigation described this process as follows: “Twitter and Instagram are filled with celebrities and health bloggers attributing their glowing appearance to eating ‘clean’… but in trying to emulate them, I became so obsessed with eating the perfect diet that it took over my life” (p. 598) [6].

The present investigation is limited by our use of a cross-sectional survey design with a limited number of questions. We are thus unable to comment on how perceptions of “clean” diets are linked with actual dietary behaviors. Further, we defined “clean” diets mid-way through the survey (to assist those who had never heard of the term in completing the survey), and although we used both negative (“strict avoidance”) and positive (“natural, whole”) descriptors, our definition may have influenced willingness to try “clean” diets among participants. Moreover, although we measured definitions of “clean” eating, we did not directly assess motivational features underpinning the pursuit of “clean” diets and therefore cannot comment on the many reasons why people may pursue these diets.

Overall, our findings offer a useful glimpse into perspectives on the “clean” dietary trend from a large, diverse sample of young people in the U.S. Given the constantly evolving nature of dietary fads, researchers face an ongoing challenge to understand their impact. Nonetheless, future studies should assess actual dietary behaviors to investigate the potential benefits of “clean” diets and the mechanisms by which formerly healthy behaviors may devolve into disordered eating. For instance, the pursuit of “clean” diets may in some cases represent (and indeed, mask) underlying body dissatisfaction and related eating disorder symptomatology, and thus warrant further scrutiny to prevent harm. Future researchers may also seek to identify strategies for effective public health communication to mitigate the potential harms of these new waves of diets on vulnerable youth. Indeed, current findings suggest that “clean” eating holds different meanings for different people, so public health messaging must carefully consider these impressions and promote a more nuanced understanding that acknowledges the potential risks of such “wellness” oriented diet fads.

## Figures and Tables

**Table 1:** Demographic features of respondents.

Self-Reported Characteristic	*n* (%)
Gender	
Female	539 (52.8%)
Male	391 (38.3%)
Transgender	45 (4.4%)
Nonbinary	25 (2.5%)
Other	15 (1.5%)
Race	
American Indian or Alaskan Native	30 (2.9%)
Asian	143 (14%)
Black or African-American	150 (4.7%)
Native Hawaiian or other Pacific Islander	12 (1.2%)
White or Caucasian	752 (73.7%)
Other	50 (4.9%)
Hispanic or Latino	149 (14.6%)
Educational Level (self)	
8th grade or less	48 (4.7%)
Some high school	541 (53%)
High school graduate	102 (10%)
Some vocational school	2 (0.2%)
Some college	200 (19.6%)
Completed vocational school	7 (0.7%)
Completed an Associate’s degree	22 (2.2%)
Completed a Bachelor’s degree	70 (6.9%)
Some graduate school	16 (1.6%)
Completed a Master’s degree	7 (0.7%)
Some graduate training beyond a Master’s degree	2 (0.2%)
Received free or reduced school lunch in middle/high school	385 (37.7%)

**Table 2:** Awareness of “clean” eating and sources of information.

**Have You Previously Heard about “Clean” Eating?** **% (*n*)**	
Yes	54.6% (557)
No	45.4% (463)
**If So, How Did You Hear about It?** **% (*n*)**	
Source was specified (yes)	47% (480)
Social media	194/480; 40.4%
Unspecified	52.6% (102)
Instagram	22.2% (43)
Tumblr	1.0% (2)
Twitter	1.0% (2)
YouTube	10.3% (20)
Facebook	12.9% (25)
Reddit	1/5% (3)
Pinterest	5.7% (11)
Other social media	0.5% (1)
Online (unspecified)	102/480; 21.3%
Commercials	18/480; 3.8%
Blogs	13/480; 2.7%
Human contact	150/480; 31.3%
Unspecified/other	32.7% (49)
Parent	14.0% (14)
Peer	44.7% (67)
Sibling	4.0% (6)
Health professional	8.0% (12)
Educator	1.7% (4)
Exercise/fitness/trainer	1.7% (4)
Educational resources	3/480; 0.6%
Schools	28/480; 5.8%
TV programs	25/480; 5.2%
Magazines	23/480; 4.8%
Media (other/unspecified)	40/480; 8.3%

Note: Numbers indicate the percentage of respondents whose answers reflected the theme.

**Table 3:** Definitions of “clean” eating, opinions, and perceptions of healthiness vs. harm.

Theme % (*n*)	Code Definition	Sample Responses
**How would you define “clean” eating?**		
Perceived healthiness 38.7% (382)	Response notes that “clean eating” is “healthy” or “good for you” or alternately involves avoiding things that are seen as “unhealthy”.	“Eating healthy” “Eating foods based on dietary guidelines. A lot of green foods, close to no junk food, eating foods that are organic and a lot of vegetables and fruits.”
Skepticism 0.6% (6)	Response indicates a critical or skeptical perspective of “clean eating”.	“Eating foods perceived to be healthier than other foods (fresher, less processed, colorful but only naturally colorful) even when there is no scientific evidence that these foods are healthier” “Clean eating is just any eating or food that is healthier sounding, but might not actually be healthy…”
Detox/cleanse 2.0% (20)	Response notes that “clean eating” involves purifying, de-toxification, or cleansing the body in some way.	“…maybe it’s some sort of cleanse.” “A way to cleanse your body and eat healthy”
Environment/ sustainability 4.2% (41)	References environment and sustainability benefits to “clean eating”.	“…no waste other than compostable items” “Only eating food grown organically and sustainably”
Non-diet-related 2.2% (22)	Response includes other types of “cleanliness” that are unrelated to diet/nutrition or environment.	“Eating with rinsed or sanitized foods” “Not eating really messy”
Limit/avoid/restrict foods 29.7% (293)	Response uses dieting/restricting terminology such as “avoid” “restrict” “only eat if…” and so forth.	“Eating only things that are good for you” “Healthy eating, no fats no fried food, no juices” “Eating nothing but veggies and fruit, very low or no carb. No sugar or fat”
Calorie reduction/weight loss 0.9% (9)	Definition explicitly notes fewer calories and/or weight loss as part of “clean eating.”	“Avoiding unhealthy food in an explicit way to lose weight” “…cutting off calories in order to get a better diet.”
Body functionality/ mindfulness 2.6% (26)	Response identifies being in tune with one’s bodily needs/cues/functions and/or being mindful/present in the moment in one’s approach toward eating.	“…eating healthy foods in order to take care of your body.” “Eating mindfully”
Processed/whole foods 39.7% (391)	Response comments on processed/unprocessed foods, raw foods, natural foods, or whole foods in some manner (including use of artificial colors/flavors).	“Eating things without preservatives or artificial colors or flavoring.” “Eating food that has not been processed/minimally processed.” “avoiding processed foods”
Genetically modified (GMO)/organic 13.4% (132)	Response comments on GMO or organic foods in some manner.	“Eating organic” “It sounds like a diet in which you only eat organic foods.”
Other farming practices 1.8% (18)	Response comments on pesticides, cage-free/free-range, or other farming practices not noted above.	“…food that has no pesticides on it” “Eating healthy things with no chemicals”
**“Clean” eating usually means eating natural, whole foods, and strictly avoiding processed foods. Knowing that, what is your opinion of “clean” eating?**		
Comments on “healthiness” “Healthy” 88.3% (211) “Unhealthy” 4.6% (11) “Both healthy and unhealthy” 7.1% (17)	Response specifically indicates that it is unilaterally a healthy or unhealthy strategy, or, suggests both healthy and unhealthy features.	Healthy: “…a healthy eating style” Unhealthy: “I do not support it, because it encourages restrictive eating and cutting out food groups, two harmful practices that can lead to eating disorders.” Both Healthy and Unhealthy: “It can be good for your general health, but when taken too far it can become dangerous”
Comments on “wellbeing” “Positive” 75.0% (129) “Negative” 6.4% (11) “Both positive and negative” 18.6% (32)	Response is characterized by positive or negative views on the impact of “clean” eating on one’s general well-being, or, suggests both positive and negative perspectives.	Code 1: “…could definitely benefit you in a plethora of ways” Code 2: “it sounds bothersome, having to know what foods are okay to eat and what aren’t, according to that definition. And I’m not sure what benefits it would provide, it sounds like it’s not worth it.” Code 3: “I think clean eating can be good, but some people go overboard with it”
Ambiguous “Positive” 88.0% (345) “Negative” 12.0% (47)	Response that indicates a generally positive or negative impression but does not offer a specific reason and does not refer to any component of well-being or health.	Positive: “Seems like it would be good” Negative: “Not good”
Ambivalent 7.5% ( 72)	Response offers ambivalent or neutral impressions toward “clean” eating.	“I believe it is the persons [sic] choice whether or not they want to eat natural foods however personally, I will eat whatever foods processed or not.” “I think that it makes sense if some people want to live that way but processed foods aren’t that bad”
Consideration of cost/finances 14.2% (136)	Response highlights the financial aspects of pursuing “clean eating”.	“it’s a smart choice for those who have the time and money for those options” “I think that it’s peoples’ choice to do what they want. A lot of people can’t afford clean eating, and a lot of stuff that is bad for you is tasty.”
Consideration of practicality/feasibility 15.3% (147)	Response highlights practical implications (i.e., lack of feasibility) regarding the pursuit of “clean” eating.	“…it might be hard. It sounds ideal, but I’m not sure I’d be able to completely eradicate processed foods.”
Consideration of environment/ sustainability 1.2% (12)	Response comments on environment/sustainability components to “clean” eating.	“I think it’s good for your body and the environment.” “It’s good considering how much processed foods contribute to our climate change…”
**Do you think “clean” eating is healthy or harmful? Tell us why.**		
Healthy 70.8% (669)	Answer specifies that “clean eating” is strictly healthy or good for one’s body.	“Healthy! Does nothing but good for your body because you’re only putting good stuff in” “Healthy because you wouldn’t be eating addictive chemicals”
Harmful 5.8% (55)	Answer specifies that “clean eating” is strictly unhealthy or harmful.	“I think it’s harmful because people might not be eating enough. My friends are eating similar to this and are not eating enough and worry too much about food.” “Harmful….I believe that it is a very disordered way of eating that puts foods in categories of only “clean” i.e., good or “dirty” i.e., bad…”
Both, healthy and harmful 18.0% (170)	Answer specifies that “clean eating” is both healthy and harmful in the context of one’s health.	“Healthy but can be harmful if not getting all your carbs and food groups in per day” “Healthy when used as a guideline. It’s unhealthy when you obsess over food and count calories”
Neither healthy nor harmful 2.3% (22)	Answer specifies that “clean eating” is neither healthy nor unhealthy.	“I think neither, because I know nothing about it. The words make me think *healthy* but I don’t form opinions without science to back it up.” “It’s neither, you can be healthy and not clean or vice versa”
Other 1.7% (16)	Answer does not address healthiness/unhealthiness of “clean eating”.	“I think it’s harmful for the environment because the energy needed to produce Whole Foods may offset the pollution saved from having this lifestyle”
Why? Restrictive behavior. 8.6% (81)	Answer notes that clean eating can involve harmful dietary behaviors such as rigidly following a diet and/or restricting one’s food intake.	“Harmful, because it encourages harmful practices like restrictive eating.” “I think it is harmful because it encourages people to unduly restrict their diets and to see certain foods as “bad.” This is also a hallmark of disordered eating.”
Why? Negative emotion. 2.3% (22)	Answer notes that “clean eating” promotes feelings of guilt/shame or other negative emotions about eating behavior.	“Harmful because it perpetuates the myth that people need to have bad foods and good foods which is part of diet culture” “…Lastly, having a healthy relationship with food is more important that eating 100% clean. The idea that some foods are bad is harmful. Nobody should feel guilty about eating.”
Why? Rigid cognition. 2.0% (19)	Answer notes that “clean eating” promotes thinking obsessively and/or ruminating/worrying about food, spending too much time thinking/planning food.	“…It’s unhealthy when you obsess over food and count calories” “Harmful because it’s not moderation and an obsession of you don’t get the right nutrients”
Why? Eating disorder risk. 1.6% (15)	If the answer goes beyond behavioral restriction, negative emotion, and/or cognitive preoccupation, and *explicitly* specifies risk of eating disorders.	“Could go either way. It could lead to anorexia or other eating disorders.” “Mostly healthy. but some people might push this diet to the extreme and not understand the full definition. For example, if they think that clean eating means eating less food, this could turn into an eating disorder”

Note. Due to non-mutually exclusive coding, percentages in the table sum to greater than 100%.
